# Cognitive-Behavioral Predictors of Individual Variability of Functional Connectivity in Healthy Young Adults

**DOI:** 10.21203/rs.3.rs-9683033/v1

**Published:** 2026-06-07

**Authors:** Colin Hawco, Julia Gallucci, Justin Ng, Maria T. Secara, Ju-Chi Yu

**Affiliations:** Centre for Addiction and Mental Health; Centre for Addiction and Mental Health; Centre for Addiction and Mental Health; Centre for Addiction and Mental Health; Centre for Addiction and Mental Health

## Abstract

While stable patterns of fMRI task-evoked brain activity and functional connectivity (FC) exist at the population level, a growing body of research emphasizes that variability exists across individuals. These differences define the critical idiosyncrasies in cognition and behavior across individuals that make individuals unique. Resting-state fMRI data (60 minutes) were examined from 1012 participants from the HCP dataset of healthy adults between the ages of 22 and 37. Functional connectivity was estimated between 360 regions, and variability was defined by each individual’s mean correlational distance (MCD) to all other participants. High MCD indicated a more ‘idiosyncratic’ connectivity pattern deviating from the group pattern. Hierarchical regression was used to determine predictors of variability in FC. The base model (demographics, sleep, sex, brain volume) explained 9.22% of the variance in heterogeneity in functional connectivity. Increased variance was explained by cognition, squared cognition, and NEO personality scores, while emotional scores and fitness explained no additional variance. The final model explained 11.9% of the variance in MCD. Low MCD (i.e., being closer to average) was associated with higher BMI, greater crystalized cognitive scores, more positive emotional valence, and NEO Agreeableness. Greater variability was associated with age, brain volume (potentially a sex difference), and NEO Extroversion. The model underestimated variability in the highest MCD participants, suggesting unexplained factors in highly variable individuals. Differences were observed between males and females, and monozygotic twins showed similar variability, suggesting a genetic component. These results suggest benefits for a connectivity pattern being more similar to the group average.

## Introduction

Functional connectivity research of the human brain has led to the discovery of discrete brain networks that are reliably identifiable across the population (Gordon, Laumann, Gilmore, et al., 2017; Ji et al., 2019; Yeo et al., 2011). These brain networks subserve distinct functions and have been aligned with an evolutionary ‘gradient’ from lower-level unimodal sensory networks to higher-level multimodal cognitive networks (Huntenburg et al., 2018). While fundamental principles of brain organization exist at the population level, significant individual differences emerge in specific connectivity patterns, brain network organization, and task-evoked activity (Braga & Buckner, 2017; Gordon, Laumann, Adeyemo, Gilmore, et al., 2017; Gordon, Laumann, Gilmore, et al., 2017; Hawco et al., 2021). An individual’s functional connectivity profile contains enough unique information to be identified, similar to a fingerprint (Finn et al., 2015), and resting state functional connectivity profiles can identify individual patterns of task-evoked brain activation (Tavor et al., 2016). These differences in network organization and functional connectivity patterns are a crucial part of what makes us unique human beings with a range of cognitive abilities and personality differences.

A growing body of literature has focused on individual variability in brain function. Early work from Miller and colleagues examining memory retrieval during task functional Magnetic Resonance Imaging (fMRI) demonstrated variability across individuals related in part to different cognitive strategies (Miller et al., 2012; Miller & Van Horn, 2007). Interestingly, patterns present in the group analysis did not represent patterns at the individual level well (Miller & Van Horn, 2007). This highlights certain challenges with group averaging approaches, as the patterns observed within a group may not be generalizable to the individual level, and some individuals may exhibit significant deviations from the group norm. Looking at resting-state functional connectivity, Gordon and colleagues identified that group-level ‘patches’ (brain regions associated with specific networks) were reliably present across individuals, but the size and topography of those regions could vary substantially across individuals (Gordon, Laumann, Adeyemo, & Petersen, 2017). Recent related work has demonstrated substantial individual variability in task-evoked activity during a range of cognitive tasks, with the dominant patterns of variability falling along a ‘positive to negative’ spectrum of brain activity (Hawco et al., 2021).

Variability is also an emerging theme in psychiatric neuroscience as clinical samples are often heterogeneous (Voineskos et al., 2020). Such heterogeneity has partly contributed to inconsistent results across studies, leading to a replication crisis in psychiatric neuroscience (Nosek et al., 2022; Nour et al., 2022). As general variability also exists in the healthy population (Gordon, Laumann, Gilmore, et al., 2017; Hawco et al., 2021), and some clustering in psychiatry suggests substantial overlap in clinical samples and control populations (Hawco et al., 2019), we have begun to evaluate individual variability as a metric of interest in psychiatric samples (Gallucci, Pomarol-Clotet, et al., 2022; Gallucci, Tan, et al., 2022; Hawco et al., 2020; Secara et al., 2024). This was done by examining the mean of the correlational distances across participants in task-evoked activity or resting-state functional connectivity. Mean correlational distance (MCD) measures how similar or dissimilar a participant is to the general group pattern represented by the average, with a higher MCD indicating a more idiosyncratic/variable pattern (Hawco et al., 2020). Of note, MCD is a summary metric of the whole-brain pattern; high variability measured by MCD in a given individual may represent a unique pattern of ‘deviation’ within that individual. In this way, MCD moves away from linear approaches which assume the same brain metrics related to a given behavioral variable are the same across individuals, and acknowledges that wide variability seen across individuals (Hawco et al., 2021; Westlin et al., 2023). MCD allows two individuals to deviate from the group pattern based on unique differences in brain function. High MCD during task fMRI has been associated with poorer performance during the N-back task in Autism Spectrum Disorder (Hawco et al., 2020) and in Schizophrenia Spectrum Disorders (Gallucci, Tan, et al., 2022). More recently, high MCD in functional connectivity has been related to having a schizophrenia diagnosis and worse cognition (Secara et al., 2024).

The purpose of the current study is to explore individual variability of functional connectivity as a key metric in relation to several behavioral variables in a large sample of healthy young adults. We used MCD in resting-state functional connectivity as a metric of the typicality of an individual’s connectivity profile (i.e., the higher the MCD, the more atypical the profile). We then compared this metric to several demographic and behavioral variables. As males have been reported to show greater variability in some brain metrics (Forde et al., 2020; Hodgetts & Hausmann, 2023), sex effects were explored, and a sex-stratified analysis was performed. We hypothesized that lower variability (i.e., more typical connectivity pattern) would relate to higher cognitive scores and other beneficial outcomes.

## Methods

### Sample

Resting-state fMRI, demographic, and cognitive-behavioral data were used from the Human Connectome Project (HCP) S1200 release (Glasser, Smith, et al., 2016; Van Essen et al., 2013). Only participants with complete data (e.g., all scans and cognitive-behavioral variables) and low motion (mean framewise displacement, FD, < 0.2 mm) (Power et al., 2012) were included. A total of 1012 participants (470 males, mean age = 28.7, standard deviation (SD) of age = 3.7, age range = [22, 37]) were included in this analysis. All HCP participants provided informed consent as part of the HCP protocols, and data were accessed with permission from the HCP. Use of the HCP data was approved by the NIH and the local Research Ethics Board at the Centre for Addictions and Mental Health.

### Extracting Functional Time Series

HCP included 4 resting fMRI scans of 15-minute duration (1200 volumes), split across two consecutive days (Smith et al., 2013). On each day, two scans were collected using different phase-encoding directions (left-right and right-left). For each scan, multiband slice acquisition was run using a customized 3T MRI Siemens “Skyra” scanner (TR = 720 ms, TE = 33 ms, flip angle = 52°, voxel size = 2 mm isotropic, 72 slices at multiband acceleration factor = 8, matrix = 104 × 90, FOV = 208 × 180 mm). HCP provides surface-based gray ordinate cortical preprocessed data for each scan including HCP minimal preprocessing (e.g., B0 distortion correction, co-registration to T1-weighted images, and normalization to a surface template), ICA-FIX (i.e., semi-automated ICA-based noise removal), and regression with the 24 motion parameters (Glasser et al., 2013). Further acquisition and preprocessing descriptions are found in the original Glasser et al. (2013) publication. As additional steps, we demeaned the fMRI time series within each voxel and performed global mean signal regression by removing the mean gray matter time series, using a GSR adjusted for the post-FIX results as suggested by the HCP group. This procedure avoided reintroducing noise (i.e., regressed FIX-identified noise components and motion parameters) and improved brain-behavior associations (Greene et al., 2018; J. Li et al., 2019). Voxel time series were averaged within 360 cortical regions as defined in the HCP-MMP 1.0 atlas (Glasser, Coalson, et al., 2016). Functional connectivity was calculated by Pearson’s correlation between the 4-run-concatenated fMRI time series between every pair of regions. The lower triangles of the individual connectivity matrices (representing all unique connections) were vectorized for subsequent analyses.

### Edgewise motion removal

It is known that motion can have distributed and non-linear effects on connectivity across participants (Power et al., 2012; Satterthwaite et al., 2012), and motion is related to variability as defined by our correlational distance metric (Gallucci, Pomarol-Clotet, et al., 2022; Gallucci, Tan, et al., 2022). As such, an additional step was performed by regressing out motion (FD) for each participant, separately for each connectivity edge.

### Calculating Mean Correlational Distance (MCD)

Data was stacked into a participants (n = 1012) by connections (n = 64,620) matrix from which the correlational distances between participants were calculated (correlational distance is 1 - R, the Pearson’s correlation). This generated an n × n (1012 × 1012) matrix of how similar the connectivity profiles are for each pair of participants. The average of the rows represents the MCD, a measure of individual variability (Gallucci, Pomarol-Clotet, et al., 2022; Gallucci, Tan, et al., 2022; Hawco et al., 2020; Secara et al., 2024). A high MCD indicated that participants were far from the average pattern observed across the group and represented an idiosyncratic pattern in the data (i.e., a more unique, less typical connectivity profile).

### Reliability of MCD

To assess the reliability of the MCD, we calculated connectivity matrices on runs 1 and 2 (day one scan) and separately on runs 3 and 4 (day two scan). MCD for each participant was calculated on each of these matrices, and Pearson’s correlation between each split was used to measure reliability.

### Relationships between Variability and Network Activity

Participants were divided into high- and low-variability sub-groups via a median split. For each sub-group, we examined the difference in their connectivity magnitude by computing the average strengths of within-network connectivity for each of the twelve brain networks (Ji et al., 2019). We used independent sample *t*-tests to examine the group differences (high- vs low-variability) in within-network connectivity strength, with all results’ false discovery rate (FDR) corrected for multiple comparisons. Cohen’s *d* was also reported. We also calculated the SD for each connectivity edge separately for the high- and low-variability groups. Participants in the high-variability group should exhibit higher SD, based on how the group was defined. Nonetheless, evaluating SD within the high-variability group remains useful to assess whether particular connections demonstrate a disproportionately greater degree of variability. Pearson’s correlation between the vectorized SD matrices of the high- and low-variability groups was performed to determine if the same spatial variability pattern was present in the high- and low-variability groups.

### Behavioral-Demographic Variables

Demographic variables in this data included age (in years), self-reported sex (binary male/female, as provided by HCP), body mass index (BMI), and self-identified race (White, Black, Asian, or ‘other’). Whole brain volume was included due to its effect on resting-state functional connectivity (Hänggi et al., 2014) and its relationship with cognitive performance (Gignac & Bates, 2017; Pietschnig et al., 2015), as suggested by prior work on the HCP dataset (Dubois et al., 2018). Whole brain volume was defined as the volume of all voxels that are neither background nor brainstem from the FreeSurfer Summary Statistics (FS_BrainSeg_Vol). Motion as the mean FD (Power et al., 2012) across the four runs. Several behavioral variables were explored as they related to MCD. Sleep quality was assessed by the total score from the Pittsburgh Sleep Quality Index (PSQI). We measured cognitive ability by the age-adjusted composite fluid (CogFluid) and crystalized scores (CogCrystal) from the NIH cognitive toolbox; the general/total composite score was not included due to a high correlation with the fluid composite (*r* = 0.84). We also measured emotional affect and well-being as the average of items from the NIH Emotional toolbox for positive well-being (posEM) and negative affect (negEM), and assessed personality via the five personality subscales of the NEO Five-Factor Inventory (Costa & McCrae, 1989). Physical fitness was measured by the age-adjusted strength (STR; grip test) scores and endurance (END; 2-minute walk test).

### Predictors of MCD

A hierarchical regression was used to build predictive models of MCD, identifying variables that predicted deviation from the average connectivity profile. A total of six models were sequentially built, with a change in adjusted *R*^2^ between models assessed via ANOVA. **Model 0** included an initial set of demographic and related variables, including age, sex, BMI, race (as dummy variables, coded White, Black, and Asian, with ‘other/undefined’ as the left-out variable), PSQI total score, mean FD (motion), and total brain volume. The models then sequentially included variables of interest. As we observed relationships between cognition and MCD in an analysis of controls and schizophrenia (Secara et al., 2024), and dynamic connectivity in HCP (Ng et al., 2024), cognition was a factor of particular interest and was added early. We also examined the square of the cognitive scores to consider potential non-linear effects; square was not added to other variables to avoid excessive terms in the model. Emotional/personality factors (representing more multifaceted constructs that may involve more complex relationships with functional connectivity) were assessed later in the model. The ordering of models was

### Model 1

Fluid and Crystalized composite scores

### Model 2

Squared Fluid and Crystalized composite scores, for potential non-linear effects, as cognition was a variable of specific interest.

### Model 3

Positive and negative emotional variables (posEM and negEM).

### Model 4

NEO ‘big five’ personality subscales.

### Model 5

Physical fitness variables (endurance and strength).

### Family and Twin Analysis

To assess potential genetic effects on MCD, we performed an additional analysis using twin and sibling status to examine if MCD was more similar in those with greater genetic similarity. We identified pairs of participants with varying levels of genetic relationships. As most participants had siblings in the study, and potentially multiple siblings, we simplified the siblings analysis by examining exclusive pairs (i.e. each participant could only be one sibling pair). First, we identified pairs of monozygotic (genetically identical) and removed those twins and any remaining siblings from the dataset. We then examined dizygotic twins, again removing any remaining siblings, and finally identified non-twin sibling pairs, including only one pair from each family. As there were very few remaining unmatched participants, we created non-sibling pairs by randomly matching from the whole sample pairs of participants from different families (i.e., unmatched pairs may have included individuals who were twins with another participant, 506 pairs were created). We then extracted the connectivity matrices from each participant and calculated a single correlational distance score for each pair, measuring how similar that pair was. For MCD, we created a difference score for MCD between pairs (i.e., sib1 MCD - sib2 MCD), to determine if MCD was more similar in those who were more genetically related.

## Results

### Reliability of Correlational Distance

When examining the split matrices for each participant (MCD calculated on runs 1 and 2 versus runs 3 and 4), there was a moderate correlation (R = 0.54, p < 0.001) across days. This suggests moderate reliability for the MCD for individual variability across scanning sessions. This is comparable to or superior to other fMRI metrics such as network connectivity (Gordon, Laumann, Gilmore, et al., 2017; Noble, Scheinost, et al., 2017).

### Connectivity Differences between High- and Low-Variability Participants

**Figure 1:**
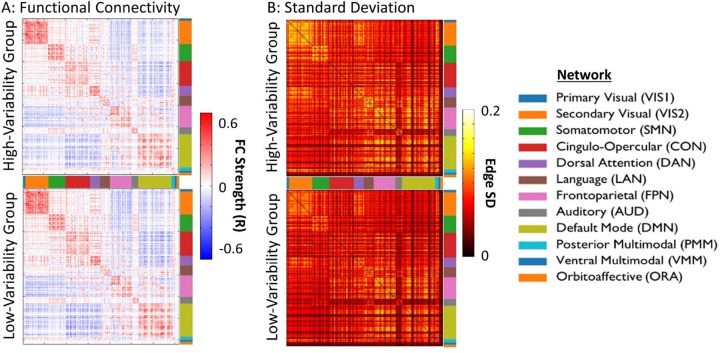
**A)** Average connectivity of those with higher variability (top) and those with lower variability (more typical; bottom), based on a median split. No apparent differences are present in the connectivity matrices; both groups show similar patterns of positive and negative correlations across networks. **B)** Standard deviation (SD) for each edge in the high-variability group (top) and low-variability group (bottom). A similar pattern is present across groups; connections showing relatively high SD in the low-variability group also show relatively high SD in the high-variability group, but with greater magnitude.

**Figure 2:**
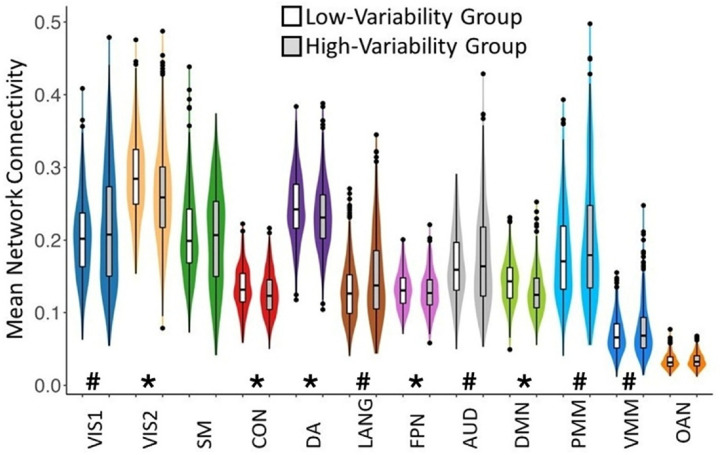
Mean within-network connectivity within the low-variability (white boxplots, left columns) and high-variability (gray boxplots) sub-groups. FDR-corrected group differences are naked with ‘*’ for high-variability > low-variability, and ‘#’ for low-variability > high-variability sub-groups. VIS1 and VIS2 = visual networks, SM = sensory-motor, CON = cingulo-opercular network, DA = dorsal attention, LANG = language network, FPN = fronto-parietal network, AUD = auditory network, DMN = default mode network, PMM = posterior multimodal network, VMM = ventral multi-modal network, OAN = orbito-affective network.

To visualize differences in connectivity between high- and low-variability participants, a median split was performed and average connectivity was plotted for each group (Fig. 1A). The results suggested that there were no systematic differences in the connectivity pattern between high- and low-variability participants. Likewise, a similar pattern of SD across edges was observed in the high- and low-variability groups (Fig. 1B). As expected, the high variability group showed overall greater SD across edges. However, the spatial pattern of this SD was very similar between the high- and low-variability groups (*r* = .99). The pattern of variability was common amongst the high and low groups, but regions that are variable across individuals were more so in the high variability groups. To determine if there may be magnitude differences in network connectivity, we compared the within-network connectivity between groups across all 12 networks (Fig. 2). The results from the two-sample *t*-tests (all significant p-values passed FDR correction) showed that the high-variability group had increased within-network connectivity compared to the low-variability group in the primary visual, t(1010) = 3.2, p = 0.002, d = 0.20, language, t(1010) = 6.2, p < 0.0001, d = 0.39, auditory, t(1010) = 2.1, p = 0.04, d = 0.13, posterior multimodal, t(1010) = 4.3, p < 0.0001, d = 0.27, and ventral multimodal networks, t(1010) = 3.3, p = 0.0008, d = 0.21. In contrast, the high variability group showed reduced connectivity in secondary visual, t(1010) = 7.5, p < 0.0001, d = 0.47, cingulo-opercular, t(1010) = 5.3, p < 0.0001, d = 0.34, dorsal attention, t(1010) = 5.5, p < 0.0001, d = 0.34, fronto-parietal, t(1010) = 2.4, p = 0.02, d = 0.15, and the default mode, t(1010) = 9.8, p < 0.0001, d = 0.62. No significant differences were present in the sensory motor, t(1010) = 1.5, p < 0.1, or orbito-affective networks, t(1010) = 1.8, p = 0.095.

### Behavioral Variables

Correlations across non-categorical demographic, cognitive, and trait variables are shown in Table 1. Many correlations were present across the predictor variables (data presented for descriptive purposes only). Fluid and crystallized cognitive composite scores were correlated with a *rho* = 0.29. Motion (mean FD) correlated with several variables, with a particularly high relationship with BMI (*rho* = 0.67), and negative correlations with cognition (*rho* = −0.13 and − 0.21 for fluid and crystalized, respectively) and endurance (*rho* = −0.29). Positive and negative emotional scores had moderate to high correlations with most NEO five-factor personality scores (rho = ± 0.3 to 0.6), except openness. Some differences across sexes were also examined (two-sample t-tests). Unsurprisingly, there were significant sex differences in Brain Volume (t(1010) = 27.1, p < 0.0001). Male participants were younger (t(1010) = 7.1, p < 0.0001), and had lower BMI (t(1010) = 2.4, p = 0.013) with lower crystalized cognition (t(1010) = 5.0, p < 0.0001) but no differences in fluid cognition (t(1010) = 0.5, p = 0.55). There was no difference in FD between males and females (t(1010) = 0.9, p = 0.36).

### Hierarchical Regression Predictors of MCD

Results of the hierarchical regression analysis are presented in Table 2. There was a significant increase in explained variance for model 1 (fluid and crystalized cognition scores), Δ*R*^2^ = 0.0090, *F(2, 987)* = 5.87, *p* = 0.00030, and model 2 (fluid and crystalized cognition squared), Δ*R*^2^ = 0.0055, *F*(2, 985) = 4.0, *p* = 0.019, a non-significant increase in Δ*R*^2^ for model 3 (emotional affect), Δ*R*^2^ = 0.0028, *F(*2,983*)* = 2.51, *p* = 0.080, a significant increase in model 4 (NEO personality), Δ*R*^2^ = 0.0091, *F(5,978)* = 3.02, *p* = 0.010, but no significant increase for model 5 (grip strength and endurance), Δ*R*^2^ = −0.0014, *F*(2, 976) = 0.24, *p* = 0.79. The significant p-values for models 1, 2, and 4 survived FDR correction. Model 4 was therefore adopted as the final model, with a final adjusted *R*^2^ of 0.119. The final model indicated that lower variability was associated with higher BMI, higher crystallized cognitive scores, greater positive affect, and higher agreeableness. Higher variability was positively associated with increased age, larger brain volume, larger squared crystalized cognition, and higher Extraversion (thus introversion with lower variability).

### Model Residuals were Non-Normal

**Figure 3:**
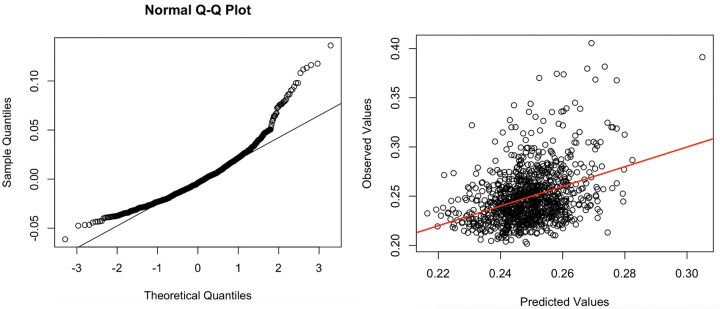
Model normal Q-Q plot (left), assessing the normality of the residuals from the final model, and predicted vs observed values (right) for Model 4. The model performed poorly at predicting variability in participants with extreme scores. This discrepancy was most pronounced in cases of high variability, where the model consistently underestimated scores.

The Q-Q model residual plot revealed non-normality in the model residuals, primarily due to poor model performance at the extremes of variability (Fig. 3). Notably, the model substantially underestimated the MCD in high variability cases, suggesting that variability in these cases is related to additional factors that are not accounted for in the current model.

### Effects of Sex

Sex was not a significant predictor in the final model (*p* = 0.28), but brain volume was highly predictive (*p* < 0.0001). As brain volume was significantly different in males and females, we hypothesized that much of the variance accounted for by sex was embedded with brain volume; the inclusion of brain volume in the final model may mask a significant sex difference. This was confirmed in that when brain volume was removed and model 4 repeated, sex was highly significant (*p* = 0.00002). We, therefore, conducted an exploratory sex-stratified analysis, repeating model 4 separately for males and females (Table 3). Model-adjusted *R*^2^ was higher for females (*R*^2^ = 0.124) than males (*R*^2^ = 0.0985). BMI and brain volume were significant predictors for both males and females, but PSQI and Fluid Cognition were significant only in males, and being black, negative emotional affect, NEO agreeableness and NEO contentedness were significant predictors only in females.

### Relationships with Predictor Variables and MCD by Network

Model 4 was rerun separately for each network, considering only within-network connectivity. Note that the number of connections used to calculate MCD for each network was substantially lower than in the above whole-brain approach. As an initial exploration, only the adjusted *R*^2^ was examined. All models produced an adjusted *R*^2^ below 0.06, except the DMN (notably the largest brain network), whose adjusted *R*^2^ was 0.143, which was higher than the whole brain model. As such, model 4 was rerun looking at MCD calculated using only the DMN. Significant predictors for the DMN MCD model were BMI (*p* = 0.0070), FD (*p* < 0.001), brain volume (*p* < 0.001), being black (*p* < 0.001), and NEO Extraversion (*p* = 0.016); NEO Agreeableness was *p* = 0.081 and NEO Neuroticism was *p* = 0.061. All other *p* > 0.10. Of note, cognitive variables were not significant (*p* > 0.40), suggesting cognition is associated with variability at the whole brain level but not at the DMN network level. On the other hand, FD was a significant predictor, suggesting MCD at the network level may be less robust to the confounding effects of motion.

### Family and Twin analysis

**Figure 4:**
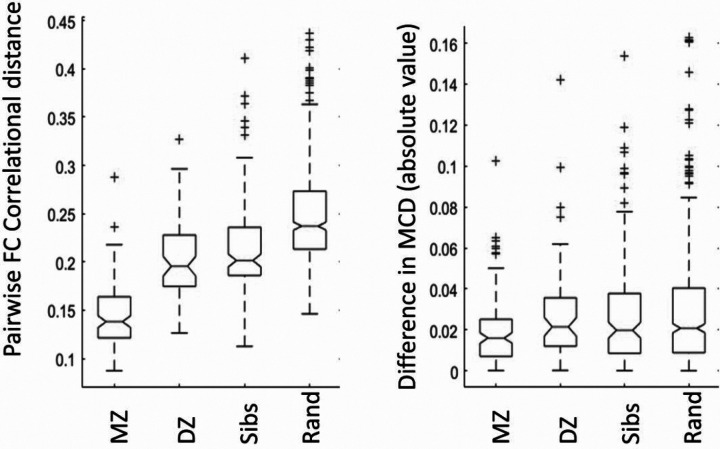
**A.** Pairwise correlational distance of functional connectivity matrices (left) between monozygotic twins (MZ), dizygotic twins (DZ), non-twin siblings (sibs), and random non-sibling pairs (Rand). MZ were more similar than DZ and sibs, which were more similar than random pairs. Pairwise absolute differences in MDC (right), showing MZ twins had more similar MCD scores than DZ, sibs, and random non-sibling pairs. DZ, siblings, and random pairs did not differ in MCD similarity.

Firstly, we examined similarity in connectivity between monozygotic twins (MZ, n = 119 pairs), dizygotic twins (DZ, n = 67 pairs), non-twin siblings (Sibs, n = 138 pairs), and randomly matched non-sibling pairs (Rand, n = 506 pairs; Fig. 4). There were significant effects of twin/family status, F(3,829) = 160, *p* < 0.001. Post hoc pairwise differences (FDR corrected) indicated MZ pairs were more similar than DZ and Sibs (both *p* < 0.001), DZ and Sibs did not differ, t(203) = 1.3, *p* = 0.18, which is not surprising given that DZ pairs are as genetically similar as non-twin sibling pairs. Random non-sibling pairs were less similar than both DZ, t(571) = 6.7, *p* < 0.001, and Sibs, t(642) = 7.0, *p* < 0.001. This demonstrates that genetically closer pairs had more similar connectivity profiles. Differences in MCD were also related to twin/family status (Fig. 4, right), one-way ANOVA, F(3,829) = 4.4, *p* = 0.0040. Post hoc t-tests indicated MZ pairs had more similar MCD than DZ (FDR corrected), t(1840 = 2.66, *p* = 0.014, Sibs, t(255) = 3.0, *p* = 0.006, and Rand, t(623) = 3.67, *p* = 0.001. There were no differences in MCD similarity between DZ, Sibs, or Rand (all p > 0.1).

## Discussion

This analysis made use of an individual variability metric, MCD, which identifies how similar a given individual’s connectivity profile is to the general group average (Hawco et al., 2020; Secara et al., 2024). MCD might be considered a measure of how ‘normative’ the connectivity patterns are for a given individual, in that it assesses whole brain connectivity in terms of how far a given individual connectivity profile deviates from the expected connectivity pattern. MCD has the advantage of assessing variability in each individual, independent of which networks or connections are different for that individual; that is, we are not assessing the effects of greater or lesser connectivity or variability within specific systems or networks, but the gestalt pattern of variability that is unique to a given individual. This makes it possible to consider idiosyncratic forms of variability, which is important as we did not find any specific networks or regions that differed more in highly variable individuals. One main finding of this study is that variability is in itself variable; each participant has a unique connectivity profile that differs from the average in very individualized ways (Gordon, Laumann, Gilmore, et al., 2017). However, having a connectivity profile that was more similar to the group average was associated with some positive outcomes such as higher cognitive scores, positive affect, and agreeableness. This result aligns with recent research demonstrating higher MCD in task fMRI among individuals with schizophrenia and bipolar disorder, where increased MCD was also associated with further negative outcomes including longer illness duration in schizophrenia and reduced cognitive performance (Gallucci, Pomarol-Clotet, et al., 2022; Gallucci, Tan, et al., 2022; Secara et al., 2024). These prior findings and the current results suggest that a typical connectivity profile offers advantages, implying that the average connectivity profile might serve as a common ‘ideal’ that the brain strives to attain during development.

Several approaches to variability have been applied to neuroimaging data, often suggesting that moderate variability is optimal (Garrett et al., 2011; S.-C. Li et al., 2006; McDonnell & Abbott, 2009). Early work on individual differences in the spatial pattern of task-evoked brain activity noted those making use of more similar cognitive strategies showed greater similarity in brain activity (Miller et al., 2012). Work from our group demonstrated that individual variability on the spatial pattern of task activity was a hallmark of task-state fMRI, with specific patterns (mainly a positive to negative axis of variability) driving differences across individuals (Hawco et al., 2019, 2021). Critically, group average analysis obscured these key sources of variability, which were also found to be related to task performance and cognition. Likewise, we found MCD to be higher during task activity (Gallucci, Pomarol-Clotet, et al., 2022; Hawco et al., 2020) and functional connectivity (Secara et al., 2024) in psychiatric samples, with variability being highly idiosyncratic rather than representing differences in discrete regions. Others have noted increased variability in global connectivity in schizophrenia (Cole et al., 2011); across studies the common pattern is higher variability in psychiatric groups, suggesting that deviation from the normative pattern is generally problematic. This growing body of work, and the current results, highlight the potential benefits of treating variability as a metric of interest, rather than unwanted variance within group average.

The final model explained less than 12% of the variance in MCD, and the Q-Q plots demonstrate that the model was particularly poor at accounting for those with higher variability. This suggests many factors remain unaccounted for. Some of this may be behavioral, as participants may engage in substantially different thought processes and in-scanner behavior. We recently used dynamic functional connectivity to demonstrate a relationship between entering stable brain states and cognition in the HCP sample (Ng et al., 2024), highlighting the importance of dynamic brain processes on individual variability. Another factor may be genetic effects, which is supported by our findings that monozygotic twins had more similar MCD scores. Undiagnosed psychiatric or neurological issues may account for further variability, and the relationship between brain volume and MCD (present in both males and females) suggests a potential link to measures of brain structure. Other unknown developmental factors (home environment, trauma exposure, education, socioeconomic factors, etc) may explain the additional variance (Farber et al., 2022; Jeong et al., 2021; Luo et al., 2022), emphasizing the need for deeper characterizations of personal history to fully understand brain function. Further work is needed to explore factors specific to those with very high variability.

Variability in resting-state fMRI has been examined in terms of the variance of the BOLD time series within regions (Garrett et al., 2013). Rather than viewing such variance as noise, variability in the time series may represent an important property of brain function (Faisal et al., 2008; Uddin, 2020), with network connectivity emerging from patterns of shared signal variance across regions (Deco et al., 2009; Mišić et al., 2011; Vakorin et al., 2011). Shared inter-regional variability has been shown to be greater within regions that are part of the same network (Baracchini et al., 2021), suggesting that shared variance is an emergent property of macro-scale brain networks. In this regard, specific forms of variability in the brain may be important characteristics associated with positive outcomes.

Temporal variability can also be explored via dynamic functional connectivity. ‘Static’ functional connectivity, as measured in our analysis, is the combination of the connectivity across different states, weighted by the time spent in those states (Cabral et al., 2017). This implies differences in the time spent in different brain states, which may be behaviorally driven by entering different mental states (e.g. boredom, introspection, etc) impacts connectivity, and by extension, MCD. This is demonstrated by relationships between different self-directed cognitive processes during the MRI scan and connectivity (Diaz et al., 2013; Pipinis et al., 2017), suggesting a portion of MCD may be driven by commonality or differences in ongoing brain state and self-directed cognitive activities during the scan. Interestingly, in our analysis of dynamic functional connectivity in HCP, participants showed that lower distances of dynamic functional connectivity patterns to the group average of specific states were related to higher generalized cognition (Ng et al., 2024).

The reliability of MCD when splitting the first and second sessions was 0.54, suggesting some proportion of MCD was variable across time or sessions, which was not accounted for in our model. Likewise, our final model only explained 12% of the variability of MCD across participants, leaving much of the variance unaccounted for. While the observed variance across days might be considered noise, it may also represent true state changes within an individual, as exemplified by work linking self-directed cognition to connectivity (Diaz et al., 2013; Pipinis et al., 2017) and differences in dynamic functional connectivity across sessions. Thus, while some differences in MCD may represent noise inherent to functional connectivity estimates, some MCD differences may represent meaningful individual differences across scanning sessions. For instance, individuals may experience greater anxiety during the MRI scan, potentially more so during the first session, and individuals may even fall asleep during the MRI (Tagliazucchi & Laufs, 2014), both of which can impact functional connectivity. We collapsed across the two MRI sessions, which may provide a more robust estimate of connectivity than data collected across a single day (Noble, Spann, et al., 2017). Another source of potential unaccounted for variability is individual differences in cortical functional organization; several studies have shown that functional regions such as the group-based parcellation used here can be variable across individuals (Dickie et al., 2018; Gordon, Laumann, Gilmore, et al., 2017; Kong et al., 2019). Better accounting for personalized connectivity and parcellations of the brain can better predict behavioral outcomes (Kong et al., 2019) and differences across clinical groups (Dickie et al., 2018) than group-based parcellations.

This study systematically evaluated individual variability in functional connectivity in a large HCP young adult cohort, taking a ‘normative’ view by examining deviation from the group’s typical pattern using MCD. The overall results suggest that having a more typical, closer-to-average connectivity profile was associated with positive outcomes, including cognition, positive affect, and agreeableness, though also with higher BMI. This suggests that the group average connectivity profile may represent an ‘ideal’ pattern, which the brain strives to achieve during development. A notable exception to this is extroversion, related to higher connectivity, which may also provide social and other benefits at the individual level. The degree to which the connection strengths fail to match the idealized average represents a broad, global marker of brain function. This, in turn, has implications for studies of mental health (Gallucci, Pomarol-Clotet, et al., 2022; Gallucci, Tan, et al., 2022; Hawco et al., 2020; Secara et al., 2024), cognitive performance (Ng et al., 2023), and other behavioral features.

## Supplementary Material

Supplementary Files

This is a list of supplementary files associated with this preprint. Click to download.

- Tables.docx

Tables 1–3 available in the Supplementary Files section.

## Data Availability

This manuscript made use of the Human Connectome Data with permission. As per NIH policies, the data cannot be shared, and must be accessed through the National Data Archive (https://nda.nih.gov/). Code used in the analysis of this manuscript are included in: https://github.com/colinhawco/HCP_Variability
